# Supplementary material for: Recombinant Adeno-Associated Viral Vectors (rAAV)-Vector Elements in Ocular Gene Therapy Clinical Trials and Transgene Expression and Bioactivity Assays
Source: Int J Mol Sci. 2020 Jun 12;21(12):4197. doi: 10.3390/ijms21124197 (PMC7352801; doi:10.3390/ijms21124197)
Supplement: Supplementary file 1 [file ijms-21-04197-s001.pdf]

Supplementary information

Title: rAAV-vector elements in ocular gene therapy clinical trials and transgene & bioactivity assays

Authors: Thilo M. Buck and Jan Wijnholds

Content:

Table S1: Description of clinical trial rAAV gene therapy products in ophthalmology / Proviral plasmids. Page 1-7

References. Page 9-13

Table S1 Description of clinical trial rAAV gene therapy products in ophthalmology

Sponsor – Product – Clinical trial phase – Gene

Achromatopsia (ACHM)

MeiraGTx – AAV-CNGA3 – I/II – *CNGA3*
Clinical trial start date (clinical trial identifier): 29-Nov-2018 (NCT03758404)
rAAV: AAV2/8.hG1.7p.h*CNGA3*co.SV40polyA
Proviral plasmid: pAAV.untoldR.ITR2.hG1.7p.h*CNGA3*co.SV40polyA.ITR2
hG1.7p=Novel synthetic cone specific promoter. Core green opsin promoter including a mutation (0.5 kb) + Locus Control Region (LCR; 1.2 kb) upstream of the red opsin gene.
Production platform: HEK293.
Citation: [1–3]

STZ Eyetrial – rAAV.h*CNGA3* – I/II – *CNGA3*
Clinical trial start date (clinical trial identifier): 20-Nov-2015 (NCT02610582)
rAAV: AAV2/8.hCAR.h*CNGA3*
Proviral plasmid: pSub.KanR.ITR2.hCARp.h*CNGA3*co.WPREm.bGHpolyA
hCAR= human cone arrestin (aka hArr3) 405 bp
CNGA3= 2085 bp full-length human CNGA3 cDNA
WPREm= WPRE is a mutated WPRE (WPREm) comprising non-expressible woodchuck hepatitis virus X protein (WHX) open reading frame (WHX OR). 543 bp
bGHpolyA= bovine growth horome 207 bp
Production platform: AAV cis (pSub-hArr3-hCNGA3-WPREm-KanR) and trans (pDP8-KanR) in HEK293 cells
Citation: [4,5]

Applied Genetic Technologies Corp. (AGTC) – AGTC-402 – I/II – *CNGA3*
Clinical trial start date (clinical trial identifier): 17-Oct-2019 (NCT02935517)
rAAV: AAV2tYF.PR1.7p.SV40 SD/SA.h*CNGA3*co.SV40polyA
Proviral plasmid: pAAV.untoldR.ITR2.PR1.7.SV40 SD/SA.h*CNGA3*co.SV40polyA.ITR2
AAV2tYF=AAV serotype 2 with surface-exposed tyrosine mutations: Y275F, Y733F, Y447F.
Production: (rHSV) complementation system in suspension-cultured baby hamster kidney (sBHK) cells
Citation: [6,7]

Applied Genetic Technologies Corp. (AGTC) – rAAV2tYF-PR1.7-h*CNGB3* – I/II – *CNGB3*
Clinical trial start date (clinical trial identifier): 9-Nov-2015 (NCT02599922)
rAAV: AAV2tYF.PR1.7.SV40 SD/SA.h*CNGB3*co.SV40polyA
Proviral plasmid: pAAV.untoldR.ITR2.PR1.7p.SV40 SD/SA.h*CNGB3*co.SV40polyA.ITR2
AAV2tYF= AAV serotype 2 with surface-exposed tyrosine mutations: Y275F, Y733F, Y447F.
Production: (rHSV) complementation system in suspension-cultured baby hamster kidney (sBHK) cells
Citation: [6–8]

MeiraGTx – either AAV-CNGB3 or AAV-*CNGA3*– I/II – *CNGB3/CNGA3*
Clinical trial start date (clinical trial identifier): 23-Dec-2016 (NCT03001310); 12-Sept-2017 (NCT03278873)
rAAV: AAV2/8.hCARp.h*CNGB3*.SV40polyA or AAV2/8.hG1.7p.h*CNGA3*co.SV40polyA
Proviral plasmid: pAAV.untoldR.ITR2.hCARp.h*CNGB3*.SV40polyA.ITR2 + pAAV.untoldR.ITR2.hG1.7p.h*CNGA3*co.SV40polyA.ITR2
Production platform: HEK293
Citation: [1–3]

Wet Age-Related Macular Degeneration (wet AMD)

Sanofi Genzyme – AAV2-s*FLT01* – I/II
Clinical trial start date (clinical trial identifier): 3-Dec-2009 (NCT01024998)
rAAV: rAAV2/2.CAGp.s*FLT01*.bGHpolyA
Proviral plasmid: pAAVSP70.untoldR.ITR2.CBA.s*FLT01*.bGHpolyA.ITR2
CBA: hybrid CMV/CBA promoter, derived from the pDRIVE CAG plasmid (Invivogen, San Diego, Calif.; having 100% sequence homology with the pCAGGS) with an upstream extension of about 49 nucleotides

of CMV enhancer (1661 bp). CBA= proximal chicken β actin promoter and human beta-globin exon 1 and intron 1. The University of Pennsylvania considers CBA and CAGGS the same.
pAAVSP70 derived from pAV1 derived from pBR322
Citation: [9–14]

Adverum Biotechnologies – ADVM-022 - I
Clinical trial start date (clinical trial identifier): 21-Nov-2018 (NCT03748784)
rAAV: AAV2/7m8.eCMV.CMVp.s*FLT01*.hSAR.hGHpolyA
Proviral plasmid: pBAC-AAV.untoldR.ITR2.eCMV.pCMV.TLP.eMLP.SyntheticIntron.Kozak.s*FLT01*co.hSAR.hGHpolyA.ITR2
Production: Baculovirus, Sf9. (plasmids: rBAC-AAV. rBAC-RepCap)
eCMV = human early CMV enhancer
pCMV = human CMV promoter
TLP = adenovirus tripartite leader sequence
eMLP = major late promoter
SI = synthetic intron. pSI chimeric intron. 5 -donor site from the first intron of the human β-globin gene and the branch and 3 -acceptor site from the intron that lies between the leader and the body of an immunoglobulin gene heavy chain variable region [15]
s*FLT01*co = codon-optimized aflibercept (recombinant chimeric protein consisting of the vascular endothelial growth factor (*VEGFA*) binding portion of human *VEGFR-1* (domain 2) and *VEGFR-2* (domain 3 or KDR) fused to the Fc portion of human *IgG1* immunoglobulin.)
hSAR = human scaffold attachment region
Production: baculovirus expression system in Sf9 cell
Citation: [15–17]

Oxford BioMedica – OXB-201 / RetinoStat – I
Clinical trial start date (clinical trial identifier): 23-Feb-2011 (NCT01301443); 5-Sept-2012 (NCT01678872)
Lenti: EIAV.CMVp.hEndo.IRES.hAngio.WPRE.LTR
Citation: [18]

Lions Eye Institute (Perth, Western Australia, Australia) / Adverum Biotechnologies – rAAV.s*Flt-1* – I/II
Clinical trial start date (clinical trial identifier): 19-Dec-2011 (NCT01494805)
rAAV: AAV2/2.CMVp.chimericIntron.s*Flt-1*.SV40polyA
Proviral plasmid: pSSV9.untoldRAmpR.ITR2.CMV.ChimericIntron.sFlt-1.SV40polyA.ITR2
sFlt-1 = full-length soluble fms-like tyrosine kinase 1 (non-membrane associated splice variant of VEGF receptor 1 [s*VEGFR-1*])
Citation: [19–22]

Hemera Biosciences – AAVCAGs*CD59* (HMR59) – I
Clinical trial start date (clinical trial identifier): 13-Jul-2018 (NCT03585556)
rAAV: AAV2/2.CAGp.s*CD59*.hGHpolyA
Proviral plasmid: pUC.untoldR.MCS(Stratagene).ITR2.CAG.s*CD59*.hGHpolyA.ITR2
sCD59 = soluble CD59 antigen binds C5b678 terminal complement protein complex and prevents incorporation of multiple C9 molecules (blockage of the Membrane attack complex [MAC] in the alternative complement cascade/pathway)
Citation: [23,24]

Regenxbio – RGX-314 – I
Clinical trial start date (clinical trial identifier): 28-Feb-2017 (NCT03066258)
rAAV: AAV2/8.ITR.CB7p.*aVEGFAfabH*.(F)/F2A.*aVEGFfabL*.rβ-globin-polyA.ITR
Proviral plasmid: pAAV.untoldR.ITR2.CAG/CB7.*aVEGFAfabH*.(F)/F2A.*aVEGFfabL*.rβ-globin-polyA.ITR2
aVEGFAfabH = anti-VEGFA Heavy chain
self-cleaving furin (F)/F2A linker
aVEGFAfabL = anti-VEGFA Light chain
rβ-globin-polyA = rabbit β-globin polyA
Citation: [25]

Dry Age-Related Macular Degeneration (dry AMD) incl. Geographic Atrophy

Hemera Biosciences – AAVCAGsCD59 (HMR59) – I
Clinical trial start date (clinical trial identifier): 9-May-2017 (NCT03144999)
rAAV: AAV2/2.CAGp.s*CD59*.hGHpolyA
Proviral plasmid: pUC.untoldR.MCS(Stratagene).ITR2.CAG.s*CD59*.hGHpolyA.ITR2
sCD59 = soluble CD59 antigen binds C5b678 terminal complement protein complex and prevents incorporation of multiple C9 molecules (blockage of the Membrane attack complex [MAC] in the alternative complement cascade/pathway)
Citation: [23,24]

Gyroscope Therapeutics – GT005 – I/II
Clinical trial start date (clinical trial identifier): 19-Feb-2019 (NCT03846193)
rAAV: rAAV2/2.CBAp.h*CFI*co.WPRE.bGHpolyA
Proviral plasmid: pBR322.UntoldR.ITR2.CBA.*CFI*.WRPE.bGHpolyA.ITR2
hCFIco= human complement factor I codon-optimized (C3b/C4b inactivator). Increases the level of C3b-inactivating and iC3b-degradation activity in the RPE
Citation: [26,27]

Choroideremia

Biogen / Nightstar Therapeutics / University of Oxford (Oxford, OFE, UK) / University of Miami (Miami, FL, USA) / University of Alberta (Edmonton, AB, Canada) / STZ eyetrial (Tübingen, BW, Germany) – rAAV2-REP1 – I/II; II – *CHM*
Clinical trial start date (clinical trial identifier): 28-Oct-2011 (NCT01461213); 4-Mar-2014 (NCT02077361); 3-Apr-2015 (NCT02407678); 17-Sept-2015 (NCT02553135); 2-Feb-2016 (NCT02671539); 25-Apr-2018 (NCT03507686); 12-Apr-2018 (NCT03496012); 12-Jul-2018 (NCT03584165);
rAAV: rAAV2/2.CBA.rabbit β-globin SD/SA.h*CHM*.WPRE.bGHpolyA
Proviral plasmid: pAAV.untoldR.ITR2.CBA.rabbit β-globin SD/SA.h*CHM*.WPRE.bGHpolyA.ITR2
Production: Triple co-transfection of HEK293 cells (AAV2 rep-cap helper plasmid; adenovirus helper plasmid containing E2A, E4ORF6, and VA RNA ORFs; pAAV)
Citation: [28–30]

Spark Therapeutics – AAV2-REP1 – I/II – *CHM*
Clinical trial start date (clinical trial identifier): 19-Jan-2015 (NCT02341807)
rAAV: AAV2/2.CBAp.h*CHM*.bGHpolA
Proviral plasmid: pAAV.Stuffer.KanR.ITR2.CBA.h*CHM*.bGHpolA.ITR2
Citation: [31]

Leber Congenital Amaurosis

Allergan / Editas Medicine Inc– AGN-151587 (EDIT-101) – I/II – LCA10 – *CEP290*
Clinical trial start date (clinical trial identifier): 13-Mar-2019 (NCT03872479)
rAAV: AAV2/5.U6.CEP290gRNAs323/.U6.CEP290gRNAs64.hGrk1.Kozak.SV40 SA/SD.*SaCas9*.NLS.spA
Proviral plasmid: pAAV.untoldR.ITR2.U6.CEP290gRNAs323/.U6.CEP290gRNAs64.hGrk1p.Kozak.SV40 SA/SD.*SaCas9*.NLS.SyntheticPolyA.ITR2
CEP290 rescue= in intron 26 of CEP290 (IVS26 c.2991+1655 A>G) gene editing (removal) of the mutation (p.Cys998X)
Modified synthetic polyA sequence: (bold+underlined=synthetic polyA sequence. 83% homology. 60 bp)
TAGC**<u>AATAAAGGATCGTTTATTTTCATTGGAAGCGTGTGTTGGTTTTTTG</u>**ATCAGGCGCG
Production: HEK293
Citation: [32,33]

ProQR – QR-110 – I/II & II/III – LCA10 – *CEP290*
AON technology. p.Cys998X mutation correction on mRNA level
Clinical trial start date (clinical trial identifier): 20-Sept-2018 (NCT03140969); 12-Apr-2019 (NCT03913143); 2-Aug-2019 (NCT03913130)
Citation: [34]

Genzyme / Sanofi - SAR439483 – I/II – *GUCY2D*
Clinical trial start date (clinical trial identifier): 18-Apr-2019 (NCT03920007)
rAAV: AAV2/5.hGRK1p.SV40 SA/SD.*GUCY2D*.bGHpolyA
Proviral plasmid: pAAV.puromycinR.ITR2.hGRK1p.SV40 SA/SD.*GUCY2D*.bGHpolyA.ITR2
Production platform: HeLaS3
Citation: [35,36]

Spark Therapeutics – AAV2-*RPE65* (h*RPE65*v2) – III – *RPE65* *FDA & EMA approved
Clinical trial start date (clinical trial identifier): 15-Aug-2007 (NCT00516477); 24-Sep-2010 (NCT01208389); 26-Mar-2018 (NCT00999609); 24-Jul-2018 (NCT03597399); 27-Jul-2018 (NCT03602820);
rAAV: AAV2/2.CBAp.h*RPE65*.bGHpolA
CBA: hybrid CMV/CAG promoter, derived from the pDRIVE CAG plasmid (Invivogen, San Diego, Calif.; having 100% sequence homology with the pCAGGS) with an upstream extension of about 49 nucleotides of CMV enhancer (1661 bp). CBA= proximal chicken β-actin promoter and human β-globin exon 1 and intron 1. The University of Pennsylvania considers CBA and CAGGS the same.
Proviral plasmid: pAAV.Stuffer.KanR.ITR2.CBA.h*RPE65*.bGHpolA.ITR2
Citation: [37]

Applied Genetic Technologies Corp (AGTC) / Hadassah Medical Organization (Jerusalem, Israel) / University of Pennsylvania (Pennsylvania, USA) – AAV2-CBSB-*RPE65* – I + I/II – *RPE65*
Clinical trial start date (clinical trial identifier): 1-Jun-2007 (NCT00481546); 13-Jan-2009 (NCT00821340); 10-Sept-2008 (NCT00749957);
rAAV: AAV2/2.CB-SBp.h*RPE65*.SV40polyA
Proviral plasmid: pAAV.KanR.ITR2.CB-SBp.h*RPE65*.SV40polyA.ITR2
CB-SB: 152 bp-shortened CBA promoter on CMV enhancer 5' end
Production: HEK293 cells, two-plasmid system
Citation: [38,39]

MeiraGTx – AAV2/5-OPTI*RPE65* – I/II - *RPE65*
Clinical trial start date (clinical trial identifier): 27-Okt-2016 (NCT02946879) 24-May-2016 (NCT02781480)
rAAV: AAV2/5.NA65p.SV40Intron.h*RPE65*co.SV40polyA
Proviral plasmid: pAAV.untoldR.ITR2.NA65p.SV40Intron.h*RPE65*co.SV40polyA.ITR2
NA65p= optimized human RPE65 promoter stronger than CBA
Production: HEK293T cells, three-plasmid system
Citation: [40]

Nantes University Hospital (Nantes, France) – rAAV-2/4.*hRPE65*– I/II - *RPE65*
Clinical trial start date (clinical trial identifier): 21-Dec-2011 (NCT01496040)
rAAV: AAV2/4.hRPEp.*hRPE65*.bGHpolyA
Proviral plasmid: pAAV.untoldR.ITR2.hRPE65p.h*RPE65*.bGHpolyA.ITR2
Production: HEK293T cells, two-plasmid system (pDP4-Kana helper plasmid)
Citation: [41]

University College (London, UK) – tgAAG76 (rAAV 2/2.hRPE65p.h*RPE65*) – I/II - *RPE65*
Clinical trial start date (clinical trial identifier): 26-Mar-2008 (NCT00643747)
rAAV: AAV2/2.hRPE65p.hRPE65.bGHpolyA
Proviral plasmid: pAAV.untoldR.ITR2.hRPE65p.h*RPE65*.bGHpolyA.ITR2
hRPE65p= 1.6 kb human RPE65 promoter
Production: B50 cell line utilizing helper adenovirus
Citation: [42]

Leber Hereditary Optic Neuropathy (LHON)

GenSight Biologics – GSO10 – III – G11778A mtDNA
Clinical trial start date (clinical trial identifier): 17-Feb-2014 (NCT02064569); 12-Jan-2016 (NCT02652780); 12-Jan-2016 (NCT02652767); 26-Sept-2017 (NCT03293524); 23-Jan-2018 (NCT03406104); 17-Sep-2018 (NCT03672968)
rAAV: AAV2/2.*ND4*

Proviral plasmid: pAAV2.untoldR.ITR.CMVp.HBB2.MTS-COX10.h*ND4*.3'COX10.ITR<br>HBB2=human β-globulin intron<br>Production: HEK293 cells<br>Citation: [43–46]
**University of Miami (Miami, FL, USA) – scAAV2-P1*ND4*v2 – I – G11778A mtDNA**<br>Clinical trial start date (clinical trial identifier): 11-Jun-2011 (NCT02161380);<br>rAAV: scAAV2/2-tyF.smCMVp. ATP1mt(MTS).h*ND4*.bGHpolA<br>Proviral plasmid: pBS.UntoldR.ITR2Δ.Sc-trs.smCMVp.ATP1mt(MTS).h*ND4*.WPRE.bGHpolA .ITR2 (P1ND4v2)<br>smCBA= a truncated chimeric CMV/CBA promoter (953 bp)<br>MTS=ATP1-based mitochondrial targeting sequence<br>AAV2-tyF= AAV2 mutated capsids Y444F+Y500F+Y730F<br>Production: HEK293 cells<br>Citation: [47,48]
**Huazhong University of Science and Technology (Huazhong, Hubei Sheng, China) – rAAV2-*ND4*– not applicable – G11778A mtDNA**<br>Clinical trial start date (clinical trial identifier): 28-Dec-2010 (NCT01267422); 15-May-2017 (NCT03153293); 9-Feb-2018 (NCT03428178)<br>rAAV: AAV2/2.CMVp.COX10(5'UTR).*ND4*.COX10(3'UTR).bGHpolyA<br>Proviral plasmid: pSNAV.neoR.ITR2.CMV.COX10(5'UTR).*ND4*.COX10(3'UTR).bGHpolyA.ITR2<br>COX10(5'UTR)= mitochondrial targeting sequence<br>Production: HEK293 + HSV1-rc/ΔUL2. Beijing FivePlus Molecular Medicine Institute<br>Citation: [49–51]
**Retinitis Pigmentosa**
**Allergan / RetroSense Therapeutics – RST-001 – I/II – advanced RP**<br>Clinical trial start date (clinical trial identifier): 22-Sep-2015 (NCT02556736)<br>rAAV: AAV2/2.CBAp.*Chop2/ChR2*.WPRE.bGHpolyA<br>Proviral plasmid: pAAV.untoldR.ITR2.CBAp.*Chop2/ChR2*.WPRE.bGHpolyA.ITR2<br>Chop2/ChR2= Microbial type rhodopsins. Light-gated cation-selective membrane channel rhodopsin-2<br>CBA= hybrid CMV/CBA promoter, derived from the pDRIVE CAG plasmid (Invivogen, San Diego, Calif.; having 100% sequence homology with the pCAGGS) with an upstream extension of about 49 nucleotides of CMV enhancer (1661 bp). THE CBA= proximal chicken β actin promoter and human beta-globin exon 1 and intron 1. The University of Pennsylvania considers CBA and CAGGS the same.<br>Production: HEK293. Citation: [12,52–54]
**jCyte, Inc – jCell – I, II –RP**<br>Clinical trial start date (clinical trial identifier): 19-Dec-2014 (NCT0232081)2; 5-Mrt-2017 (NCT03073733)<br>Single intravitreal injection of 0.5 - 3.0 million human retinal progenitor cells (hRPC)<br>Citation: [55,56]
**ReNeuron Limited – hRPCRP– I/II –RP**<br>Clinical trial start date (clinical trial identifier): 8-Jun-2015 (NCT02464436)<br>Participants will undergo vitrectomy surgery and subretinal implantation of human retinal progenitor cells (hRPC) in the study eye.
**GenSight Biologics– rAAV2.7m8-CAG-*ChrimsonR-tdTomato* (GS030) – I/II – non-syndromic**<br>Clinical trial start date (clinical trial identifier): 31-Oct-2017 (NCT03326336)<br>rAAV: rAAV2/7m8.CAGp.ChrimsonR-tdTomato.bGHpolyA<br>Proviral plasmid: pAAV.KanR.ITR2.CAG.*ChrimsonR-tdTomato*.bGHpolyA.ITR2<br>AAV2 7m8= AAV serotype 2 mutated capsid variant AAV2~^{588}LALGETTRP. No sialic acid dependence. Lower heparin affinity<br>Expression of Channelrhodopsin in retinal ganglion cells<br>Citation: [57–59]

Bionic Sight LLC / Applied Genetic Technologies Corp (AGTC) – BSO1 – I/II
Clinical trial start date (clinical trial identifier): 06-Feb-2020 (NCT04278131)
rAAV: AAV(untold).untold.*ChronosFP*(*Chr90*-fluorescent protein).untold
Proviral plasmid: untold
Expression of channelrhodopsin variant chronos (fast acting) in retinal ganglion cells
Citation:

Horama SA – AAV2/5-h*PDE6B* – I/II – *PDE6B*
Clinical trial start date (clinical trial identifier): 1-Nov-2017 (NCT03328130)
rAAV: rAAV2/5.hGRK1p.h*PED6B*.bGHpolyA
Proviral plasmid: pAAV.untoldR.ITR2.hGRK1.h*PED6B*.bGHpolyA.ITR2
Citation: [60,61]

King Khaled Eye Specialist Hospital (Riyadh, Saudi Arabia) – rAAV2-VMD2-h*MERTK*– I/II – *MERTK*
Clinical trial start date (clinical trial identifier): 30-Nov-2011 (NCT01482195)
rAAV: rAAV2/2.hVMD2p.SV40 SD/SD.h*MERTK*.SV40polyA.bGHpolyA
Proviral plasmid: pTR.ColE1ori.untoldR.F1(+)ori.ITR2.hVMD2p.SV40 SD/SD.h*MERTK*.SV40polyA.bGHpolyA.ITR2
hVMD2p= human VMD2 promoter (- 585 to + 38 bp region. 623 bp)
Production: HEK293 co-transfection (pTR-VMD2-hMerTK and pDG-KanR)
Citation: [62]

Novartis – CPK850 AAV8 – I/II – *RLBP1*
Clinical trial start date (clinical trial identifier): 15-Dec-2017 (NCT03374657)
rAAV: scAAV2/8.sRLBP1p.modSV40.h*RLBP1*.SV40polyA
Proviral plasmid: pAAV.untoldR.ITR2Δ.sRLBP1p.modSV40 SA/SD.h*RLBP1*.SV40polyA.ITR2
modSV40 SA/SD= modified SV40 splice acceptor/donor intron, 157 bp in length, nucleotides 502–561 and 1,410–1,497 of SV40 genomic sequence (NC_001669.1) + connecting sequence CGGATCCGG between two fragments.
Production: AAV293 in CellSTACK (HEK293 subclone; Stratagene). Triple-plasmid transfection (pHelper, pRep2Cap8, pAAV)
Citation: [63,64]

Applied Genetic Technologies Corp. (AGTC)/Biogen-rAAV2tYF-GRK1-*RPGR* (AGTC-501/BIIB088)-I/II-*RPGR*
Clinical trial start date (clinical trial identifier): 20-Oct-2017 (NCT03316560)
rAAV: AAV2/2tYF.hGRK1p.SV40 SA/SD.h*RPGR1*co-ORF15.SV40polyA
Proviral plasmid: pAAV.untoldR.ITR2.hGRK1p.SV40 SA/SD.h*RPGR1*co-ORF15.SV40polyA.ITR2
hGRK1p= 292 bp human GRK1 promoter (positions 1793-2087)
SV40 SA/SD= 100 bp mini SV40 splice donor/acceptor intron
h*RPGR1*co = Based on GenBank reference mRNA sequence NM_001034853 *hRPGR* isoform C. Codon optimized based on human codon usage, reduced tandem repeats, adjusted G/C content
2tYF= AAV2 triple YF mutations
Production: recombinant herpes simplex virus (HSV) complementation system in suspension-cultured baby hamster kidney (sBHK) cells
Citation: [65,66]

MeiraGTx UK II Ltd / Janssen – AAV-*RPGR* – I/II – *RPGR*
Clinical trial start date (clinical trial identifier): 17-Aug-2017 (NCT03252847)
rAAV: AAV2/5.hGRK1p.SV40 SD/SA.h*RPGR*-ORF15-L.SV40polyA
Proviral plasmid: pAAV.untoldR.ITR2.hGRK1.SV40 SD/SA.h*RPGR*-ORF15-L.SV40polyA.ITR2
hRPGR-ORF15-L= small-deletion human hRPGR-ORF15 'long form' (codons 862-988del) expresses a human RPGR-ORF15 protein of ~170 kD in size
Citation: [67,68]

Nightstar Therapeutics / Biogen - AAV-*RPGR* – II/III – *RPGR*
Clinical trial start date (clinical trial identifier): 14-Apr-2017 (NCT03116113)
rAAV: AAV2/8.hGRK1p.h*RPGR*co-ORF15co.bGHpolyA

Proviral plasmid: pAAV.untoldR.ITR2.hGRK1.h*RPGR*co-ORF15.bGHpolyA.ITR2 (AAV2.CBA.eGFP cat. 7072 Vector Biolabs)
Production: HEK293T Hyperflask. Two-plasmid co-transfection: pDP8.ape (PlasmidFactory) + pAAV
Citation: [69,70]

Usher syndrome

ProQR - QR-421a (STELLAR) – I/II – USH2A Exon 13
Clinical trial start date (clinical trial identifier): 19-Dec-2018 (NCT03780257)
RNA therapies antisense oligonucleotide exon-13 skipping
Citation: [34]

Sanofi / Oxford Biomedical - EIAV-CMV-*MYO7A* (UshStat) – I/II – Usher syndrome type 1B / MYO7A
Clinical trial start date + #: 6-Jan-2012 (NCT01505062); 17-Feb-2014 (NCT02065011)
Lenti: EIAV.SIN-LTR.NeoR.CMVp.h*MYO7A*.WPRE.SIN-LTR
Production: HEK293T co-transfection pHCMVG, pESynGP, pONY8-EIAV.CMV. h*MYO7A*.LTR (based on pLG338-SPORT.
Citation: [71–73]

Stargardt disease

Sanofi / Oxford Biomedical - EIAV-ABCA4 (SAR422459) – I/II – *ABCA4*
Clinical trial start date (clinical trial identifier): 7-Jun-2011 (NCT01367444); 29-Nov-2012 (NCT01736592)
Lenti: EIAV.CMVp.ABCA4.LTR
Plasmid: pONY8-EIAV.SIN-LTR.NeoR.CMVp.h*ABCA4*.SIN-LTR
Production: HEK293T co-transfection pHCMVG, pESynGP, pONY8-EIAV.CMV.ABCA4.LTR (based on pONY4.0Z and pLG338-SPORT).
Citation: [72–74]

X-linked retinoschisis

Applied Genetic Technologies Corp. (AGTC) / Biogen - rAAV2tYF-CB-hRS1 – I/II – *RS1*
Clinical trial start date (clinical trial identifier): 15-Apr-2015 (NCT02416622)
rAAV: AAV2/tYF.CBAp.hRS1.WPRE.SV40polyA
Proviral plasmid: pTR-AAV.untoldR.ITR2.smCB.h*RS1*.WPRE.SV40polyA.ITR2
smCB= 953 bp chimeric CMV-chicken β-actin promoter (shortened hybrid chicken β-actin/rabbit β-globin intron)
tYF= AAV serotype 2 with surface-exposed tyrosine mutations: Y275F, Y733F, Y447F.
Production: rHSV + sBHK cells. Two-plasmid co-transfection system (pAAV, pHelper(AAV2rep.AAV2tYFCap)
Citation: [75–77]

National Eye Institute (Washington DC, USA) - AAV8-RS1 – I/II – *RS1*
Clinical trial start date (clinical trial identifier): 17-Dec-2017 (NCT02317887)
rAAV: scAAV2/8.IRBPe.hRS1p.hRS1.hBGpolyA
Proviral plasmid: pAAV.untoldR.ITR2Δ.IRBPe.hRS1p.h*RS1*.hBGpolyA.ITR2
IRBPe= interphotoreceptor retinoid-binding protein (IRBP) enhancer element
hRS1p= modified tissue-selective human retinoschisin promoter
hRS1=an intact human retinoschisin cDNA with a truncated first intron located in its authentic position between the exon 1 and 2 sequences
hBGpolyA= human β-globin 3’ UTR and polyadenylation site
Production: triple co-transfection of HEK293 cells
Citation: [78–80]

UntoldR, not reported in the primary literature

References

1. Georgiadis, A.; Matsuki, T.; Rizzi, M.; Hoke, J.; Gonzalez-Cordero, A.; Sampson, R.; Bainbridge, J.; Smith, A.; Ali, R. ARVO Annual Meeting Poster A197 Development and efficacy assessment of AAV2/8-hG1.7p.coCNGA3, a CNGA3 gene therapy vector. *Invest. Ophthalmol. Vis. Sci.* **2019**, *60*, 3426.

2. Rizzi, M.; Ali, R.; Smith, A.; Nishiguchi, K. Gene therapy to improve vision. Patent US20180030477A1 2015, 1–66.

3. Forbes, A. *United States Securities and exchange commission (SEC) Form S-1 registration statement MeiraGTx Holdings plc*; Washington, D.C, 2018;

4. Michalakis, S.; Biel, M.; Seeliger, M. Gene therapy to improve vision. Patent US20180353619A1 2016, 1–23.

5. Michalakis, S.; Mühlfriedel, R.; Tanimoto, N.; Krishnamoorthy, V.; Koch, S.; Fischer, M.D.; Becirovic, E.; Bai, L.; Huber, G.; Beck, S.C.; et al. Restoration of cone vision in the CNGA3-/- mouse model of congenital complete lack of cone photoreceptor function. *Mol. Ther.* **2010**, *18*, 2057–2063.

6. Ye, G.-J. Promoters, expression cassettes, vectors, kits, and methods for the treatment of achromatopsia and other diseases. Patent US201361824071P 2013.

7. Gootwine, E.; Ofri, R.; Banin, E.; Obolensky, A.; Averbukh, E.; Ezra-Elia, R.; Ross, M.; Honig, H.; Rosov, A.; Yamin, E.; et al. Safety and Efficacy Evaluation of rAAV2tYF-PR1.7-hCNGA3 Vector Delivered by Subretinal Injection in CNGA3 Mutant Achromatopsia Sheep. *Hum. Gene Ther. Clin. Dev.* **2017**, *28*, 96–107.

8. Ye, G.; Budzynski, E.; Sonnentag, P.; Nork, T.M.; Miller, P.E.; Sharma, A.K.; Ver Hoeve, J.N.; Smith, L.M.; Arndt, T.; Calcedo, R.; et al. Safety and Biodistribution Evaluation in Cynomolgus Macaques of rAAV2tYF-PR1.7-hCNGB3, a Recombinant AAV Vector for Treatment of Achromatopsia. *Hum. Gene Ther. Clin. Dev.* **2016**, *27*, 37–48.

9. Scaria, A. Compositions and methods for treating and preventing macular degeneration. Patent US20170007719A1 2014, 1–61.

10. Ziegler, R.J.; Lonning, S.M.; Armentano, D.; Li, C.; Souza, D.W.; Cherry, M.; Ford, C.; Barbon, C.M.; Desnick, R.J.; Gao, G.; et al. AAV2 vector harboring a liver-restricted promoter facilitates sustained expression of therapeutic levels of alpha-galactosidase A and the induction of immune tolerance in Fabry mice. *Mol. Ther.* **2004**, *9*, 231–240.

11. Laughlin, C.A.; Tratschin, J.D.; Coon, H.; Carter, B.J. Cloning of infectious adeno-associated virus genomes in bacterial plasmids. *Gene* **1983**, *23*, 65–73.

12. Bennett, J. Taking Stock of Retinal Gene Therapy: Looking Back and Moving Forward. *Mol. Ther.* **2017**, *25*, 1076–1094.

13. Pechan, P.; Rubin, H.; Lukason, M.; Ardinger, J.; DuFresne, E.; Hauswirth, W.W.; Wadsworth, S.C.; Scaria, A. Novel anti-VEGF chimeric molecules delivered by AAV vectors for inhibition of retinal neovascularization. *Gene Ther.* **2009**, *16*, 10–16.

14. Heier, J.S.; Kherani, S.; Desai, S.; Dugel, P.; Kaushal, S.; Cheng, S.H.; Delacono, C.; Purvis, A.; Richards, S.; Le-Halpere, A.; et al. Intravitreous injection of AAV2-sFLT01 in patients with advanced neovascular age-related macular degeneration: a phase 1, open-label trial. *Lancet*

(London, England) **2017**, *390*, 50–61.

15. Bothwell, A.L.M.; Paskind, M.; Reth, M.; Imanishi-Kari, T.; Rajewsky, K.; Baltimore, D. Heavy chain variable region contribution to the NPb family of antibodies: somatic mutation evident in a γ2a variable region. *Cell* **1981**, *24*, 625–637.

16. Grishanin, R.; Vuillemenot, B.; Sharma, P.; Keravala, A.; Greengard, J.; Gelfman, C.; Blumenkrantz, M.; Lawrence, M.; Hu, W.; Kiss, S.; et al. Preclinical Evaluation of ADVM-022, a Novel Gene Therapy Approach to Treating Wet Age-Related Macular Degeneration. *Mol. Ther.* **2019**, *27*, 118–129.

17. Chalberg, T.W.; Neitz, J.; Neitz, M. Compositions and methods for enhanced gene expression in cone cells. Patent US20150259395A1 2015, 1–154.

18. Parker, M.; Bellec, J.; McFarland, T.; Scripps, V.; Appukuttan, B.; Hartzell, M.; Yeager, A.; Hady, T.; Mitrophanous, K.A.; Stout, T.; et al. Suppression of neovascularization of donor corneas by transduction with equine infectious anemia virus-based lentiviral vectors expressing endostatin and angiostatin. *Hum. Gene Ther.* **2014**, *25*, 408–418.

19. Lai, C.-M.; Estcourt, M.J.; Wikstrom, M.; Himbeck, R.P.; Barnett, N.L.; Brankov, M.; Tee, L.B.G.; Dunlop, S. a; Degli-Esposti, M. a; Rakoczy, E.P. rAAV.sFlt-1 gene therapy achieves lasting reversal of retinal neovascularization in the absence of a strong immune response to the viral vector. *Invest. Ophthalmol. Vis. Sci.* **2009**, *50*, 4279–4287.

20. Rakoczy, E.P.; Lai, C.-M.; Magno, A.L.; Wikstrom, M.E.; French, M.A.; Pierce, C.M.; Schwartz, S.D.; Blumenkranz, M.S.; Chalberg, T.W.; Degli-Esposti, M.A.; et al. Gene therapy with recombinant adeno-associated vectors for neovascular age-related macular degeneration: 1 year follow-up of a phase 1 randomised clinical trial. *Lancet (London, England)* **2015**, *386*, 2395–2403.

21. Constable, I.J.; Pierce, C.M.; Lai, C.M.; Magno, A.L.; Degli-Esposti, M.A.; French, M.A.; McAllister, I.L.; Butler, S.; Barone, S.B.; Schwartz, S.D.; et al. Phase 2a Randomized Clinical Trial: Safety and Post Hoc Analysis of Subretinal rAAV.sFLT-1 for Wet Age-related Macular Degeneration. *EBioMedicine* **2016**, *14*, 168–175.

22. Constable, I.J.; Rakoczy, E.P.; Lai, C.-M.; Chalberg, T.W.J. Treatment of amd using aav sflt-1. Patent US20140341977A1 2014, 1–127.

23. Cashman, S.M.; Ramo, K.; Kumar-Singh, R. A non membrane-targeted human soluble CD59 attenuates choroidal neovascularization in a model of age related macular degeneration. *PLoS One* **2011**, *6*.

24. Kumar-Singh, R.; Leaderer, D.; Cashman, S. Compositions, kits and methods for treatment of complement-related disorders. Patent US20170209535A1 2017, 1–45.

25. Liu, Y.; Fortmann, S.D.; Shen, J.; Wielechowski, E.; Tretiakova, A.; Yoo, S.; Kozarsky, K.; Wang, J.; Wilson, J.M.; Campochiaro, P.A. AAV8-antiVEGFfab Ocular Gene Transfer for Neovascular Age-Related Macular Degeneration. *Mol. Ther.* **2018**, *26*, 542–549.

26. Groendahl, C.; Funnell, T.; Hollowood, C. Gene Therapy. Patent US20190255193A1 2015, 1–53.

27. Buchberger, A. PhD thesis: The therapeutic utility of Factor I in the treatment of complement dependent pathophysiological processes, University of Leicester, 2016.

28. MacLaren, R.E.; Groppe, M.; Barnard, A.R.; Cottriall, C.L.; Tolmachova, T.; Seymour, L.; Reed Clark, K.; During, M.J.; Cremers, F.P.M.; Black, G.C.M.; et al. Retinal gene therapy in patients with choroideremia: Initial fi ndings from a phase 1/2 clinical trial. *Lancet* **2014**, *383*, 1129–1137.

29. Jacobson, S.G.; Cideciyan, A. V; Ratnakaram, R.; Heon, E.; Schwartz, S.B.; Roman, A.J.; Peden, M.C.; Aleman, T.S.; Boye, S.L.; Sumaroka, A.; et al. Gene therapy for leber congenital amaurosis caused by RPE65 mutations: safety and efficacy in 15 children and adults followed up to 3 years. *Arch. Ophthalmol. (Chicago, Ill. 1960)* **2012**, *130*, 9–24.

30. Lam, B.L.; Davis, J.L.; Gregori, N.Z.; MacLaren, R.E.; Girach, A.; Verriotto, J.D.; Rodriguez, B.; Rosa, P.R.; Zhang, X.; Feuer, W.J. Choroideremia Gene Therapy Phase 2 Clinical Trial: 24-Month Results. *Am. J. Ophthalmol.* **2019**, *197*, 65–73.

31. Wright, J.F.; Sumaroka, M. Rab escort protein potency assay. Patent US201662418637P 2016, 1–16.

32. Maeder, M.L.; Stefanidakis, M.; Wilson, C.J.; Baral, R.; Barrera, L.A.; Bounoutas, G.S.; Bumcrot, D.; Chao, H.; Ciulla, D.M.; DaSilva, J.A.; et al. Development of a gene-editing approach to restore vision loss in Leber congenital amaurosis type 10. *Nat. Med.* **2019**, *25*, 229–233.

33. Maeder, M.L.; Bumcrot, D.A.; Shen, S. CRISPR/CAS-related methods and compositions for treating Leber's Congenital Amaurosis 10 (LCA10). Patent US10253312B 2014, 1–298.

34. Vázquez-Domínguez, I.; Garanto, A.; Collin, R.W.J. Molecular Therapies for Inherited Retinal Diseases-Current Standing, Opportunities and Challenges. *Genes (Basel).* **2019**, *10*.

35. Nass, S.A.; Mattingly, M.A.; Woodcock, D.A.; Burnham, B.L.; Ardinger, J.A.; Osmond, S.E.; Frederick, A.M.; Scaria, A.; Cheng, S.H.; O'Riordan, C.R. Universal Method for the Purification of Recombinant AAV Vectors of Differing Serotypes. *Mol. Ther. - Methods Clin. Dev.* **2018**, *9*, 33–46.

36. Boye, S.E.; Boye, S.L.; Pang, J.; Ryals, R.; Everhart, D.; Umino, Y.; Neeley, A.W.; Besharse, J.; Barlow, R.; Hauswirth, W.W. Functional and behavioral restoration of vision by gene therapy in the guanylate cyclase-1 (GC1) knockout mouse. *PLoS One* **2010**, *5*, e11306.

37. Bennett, J. Gene- and Cell-Based Treatment Strategies for the Eye. In *Essentials in Ophthalmology*; Rakoczy, E.P., Ed.; Springer, Berlin, Heidelberg: Berlin Heidelberg, 2015; pp. 9–25 ISBN 978-3-662-45187-8.

38. Banin, E.; Bandah-Rozenfeld, D.; Obolensky, A.; Cideciyan, A. V.; Aleman, T.S.; Marks-Ohana, D.; Sela, M.; Boye, S.; Sumaroka, A.; Roman, A.J.; et al. Molecular anthropology meets genetic medicine to treat blindness in the North African jewish population: Human gene therapy initiated in Israel. *Hum. Gene Ther.* **2010**, *21*, 1749–1757.

39. Jacobson, S.G.; Acland, G.M.; Aguirre, G.D.; Aleman, T.S.; Schwartz, S.B.; Cideciyan, A. V.; Zeiss, C.J.; Komaromy, A.M.; Kaushal, S.; Roman, A.J.; et al. Safety of Recombinant Adeno-Associated Virus Type 2-RPE65 Vector Delivered by Ocular Subretinal Injection. *Mol. Ther.* **2006**, *13*, 1074–1084.

40. Georgiadis, A.; Duran, Y.; Ribeiro, J.; Abelleira-Hervas, L.; Robbie, S.J.; Sünkel-Laing, B.; Fourali, S.; Gonzalez-Cordero, A.; Cristante, E.; Michaelides, M.; et al. Development of an optimized AAV2/5 gene therapy vector for Leber congenital amaurosis owing to defects in RPE65. *Gene Ther.* **2016**, *23*, 857–862.

41. Le Meur, G.; Lebranchu, P.; Billaud, F.; Adjali, O.; Schmitt, S.; Bézieau, S.; Péréon, Y.; Valabregue, R.; Ivan, C.; Darmon, C.; et al. Safety and Long-Term Efficacy of AAV4 Gene Therapy in Patients with RPE65 Leber Congenital Amaurosis. *Mol. Ther.* **2018**, *26*, 256–268.

42. Bainbridge, J.W.B.; Mehat, M.S.; Sundaram, V.; Robbie, S.J.; Barker, S.E.; Ripamonti, C.; Georgiadis, A.; Mowat, F.M.; Beattie, S.G.; Gardner, P.J.; et al. Long-term effect of gene therapy on Leber's congenital amaurosis. *N. Engl. J. Med.* **2015**, *372*, 1887–1897.

43. Cwerman-Thibault, H.; Augustin, S.; Lechauve, C.; Ayache, J.; Ellouze, S.; Sahel, J.A.; Corral-Debrinski, M. Nuclear expression of mitochondrial ND4 leads to the protein assembling in complex I and prevents optic atrophy and visual loss. *Mol. Ther. - Methods Clin. Dev.* **2015**, *2*, 15003.

44. Lemanssier, S.; Brovedani, F. *2017 Registration document including the annual financial report the management report and the corporate governance report*; Paris, France, 2018;

45. Katz, B. *Innovation Showcase - GenSight: OIS @ American Society of Cataract and Refractive Surgery (ASCRS)*; San Diego, CA, USA, 2019;

46. Biologics, G. Summary notification information format for the release of genetically modified organisms other than higher plants in accordance with article 11 of directive 2001/18/EC Available online: https://gmoinfo.jrc.ec.europa.eu/bsnifs-gmo/B-FR-13-GT05.pdf.

47. Koilkonda, R.; Yu, H.; Talla, V.; Porciatti, V.; Feuer, W.J.; Hauswirth, W.W.; Chiodo, V.; Erger, K.E.; Boye, S.L.; Lewin, A.S.; et al. LHON gene therapy vector prevents visual loss and optic neuropathy induced by G11778A mutant mitochondrial DNA: Biodistribution and toxicology profile. *Investig. Ophthalmol. Vis. Sci.* **2014**, *55*, 7739–7753.

48. Koilkonda, R.D.; Chou, T.H.; Porciatti, V.; Hauswirth, W.W.; Guy, J. Induction of rapid and highly efficient expression of the human ND4 complex I subunit in the mouse visual system by self-complementary adeno-associated virus. *Arch. Ophthalmol.* **2010**, *128*, 876–883.

49. Yang, S.; Ma, S. qi; Wan, X.; He, H.; Pei, H.; Zhao, M. jian; Chen, C.; Wang, D. wen; Dong, X. yan; Yuan, J. jia; et al. Long-term outcomes of gene therapy for the treatment of Leber's hereditary optic neuropathy. *EBioMedicine* **2016**, *10*, 258–268.

50. Wu, Z.; Wu, X.; Cao, H.; Dong, X.; Wang, H.; Hou, Y. A novel and highly efficient production system for recombinant adeno-associated virus vector. *Sci. China, Ser. C Life Sci.* **2002**, *45*, 96–104.

51. Xiaobing, W.(吴小兵); Wenhong, T.(田文洪); Zheyue, D.(董哲岳); Xiaoyan, D.(董小岩) AAV vector-based high-throughput miRNA activity detection method, and applications thereof. Patent CN102719556A 2011.

52. Zhang, Z.; Feng, J.; Wu, C.; Lu, Q.; Pan, Z.H. Targeted expression of channelrhodopsin-2 to the axon initial segment alters the temporal firing properties of retinal ganglion cells. *PLoS One* **2015**, *10*.

53. Rodrigues, G.A.; Shalaev, E.; Karami, T.K.; Cunningham, J.; Slater, N.K.H.; Rivers, H.M. Pharmaceutical Development of AAV-Based Gene Therapy Products for the Eye. *Pharm. Res.* **2019**, *36*.

54. Pan, Z.-H. AAV-mediated subcellular targeting of heterologous rhodopsins in retinal ganglion cells. Patent US9968689B2 2010, 1–101.

55. Tucker, B.A.; Park, I.-H.; Qi, S.D.; Klassen, H.J.; Jiang, C.; Yao, J.; Redenti, S.; Daley, G.Q.; Young, M.J. Transplantation of adult mouse iPS cell-derived photoreceptor precursors restores retinal structure and function in degenerative mice. *PLoS One* **2011**, *6*, e18992.

56. Wiley, L.A.; Burnight, E.R.; Deluca, A.P.; Anfinson, K.R.; Cranston, C.M.; Kaalberg, E.E.; Penticoff, J.A.; Affatigato, L.M.; Mullins, R.F.; Stone, E.M.; et al. CGMP production of patient-specific iPSCs and photoreceptor precursor cells to treat retinal degenerative blindness. *Sci. Rep.* **2016**, *6*, 22–24.

57. Douar, A.M.; Bouquet, C.; Pruneau, D.; Chavas, J.; Dalkara, D.; Duebel, J.; Benosman, R.; Chenegros, G.; Picaud, S.; Sahel, J.; et al. 268. Optogenetic Engineering of Retinal Ganglion Cells with AAV2.7m8-ChrimsonR-tdTomato (GS030-DP) Is Well Tolerated and Induces Functional Responses to Light in Non-Human Primates. *Mol. Ther.* **2016**, *24*, S106–S107.

58. Dalkara, D.; Byrne, L.C.; Klimczak, R.R.; Visel, M.; Yin, L.; Merigan, W.H.; Flannery, J.G.; Schaffer, D. V In Vivo–Directed Evolution of a New Adeno-Associated Virus for Therapeutic Outer Retinal Gene Delivery from the Vitreous. *Sci. Transl. Med.* **2013**, *5*, 189ra76--189ra76.

59. Dalkara, D.; Picaud, S.; Desrosiers, M.; Sahel, J.-A.; Duebel, J.; Bemelmans, A.; Roska, B. Promoters and uses thereof. Patent US20180355354A1 2015, 1–37.

60. Pichard, V.; Provost, N.; Mendes-Madeira, A.; Libeau, L.; Hulin, P.; Tshilenge, K.T.; Biget, M.; Ameline, B.; Deschamps, J.Y.; Weber, M.; et al. AAV-mediated gene therapy halts retinal degeneration in PDE6β-deficient dogs. *Mol. Ther.* **2016**, *24*, 867–876.

61. Petit, L.; Lhériteau, E.; Weber, M.; Le Meur, G.; Deschamps, J.-Y.; Provost, N.; Mendes-Madeira, A.; Libeau, L.; Guihal, C.; Colle, M.-A.; et al. Restoration of vision in the pde6β-deficient dog, a large animal model of rod-cone dystrophy. *Mol. Ther.* **2012**, *20*, 2019–2030.

62. Conlon, T.J.; Deng, W.-T.T.; Erger, K.; Cossette, T.; Pang, J. jing; Ryals, R.; Clément, N.; Cleaver, B.; McDoom, I.; Boye, S.E.S.L.; et al. Preclinical potency and safety studies of an AAV2-mediated gene therapy vector for the treatment of MERTK associated retinitis pigmentosa. *Hum. Gene Ther. Clin. Dev.* **2013**, *24*, 23–28.

63. Choi, V.W.; Bigelow, C.E.; McGee, T.L.; Gujar, A.N.; Li, H.; Hanks, S.M.; Vrouvlianis, J.; Maker, M.; Leehy, B.; Zhang, Y.; et al. AAV-mediated RLBP1 gene therapy improves the rate of dark adaptation in Rlbp1 knockout mice. *Mol. Ther. - Methods Clin. Dev.* **2015**, *2*, 15022.

64. Choi, V.; Bigelow, C.E.; Dryja, T.P.; Reddy POLICE, S. Viral vectors for the treatment of retinal dystrophy. Patent US9163259B2 2012, 1–173.

65. Song, C.; Conlon, T.J.; Deng, W.T.; Coleman, K.E.; Zhu, P.; Plummer, C.; Mandapati, S.; Van Hoosear, M.; Green, K.B.; Sonnentag, P.; et al. Toxicology and pharmacology of an AAV vector expressing codon-optimized RPGR in RPGR-deficient Rd9 mice. *Hum. Gene Ther. Clin. Dev.* **2018**, *29*, 188–197.

66. Beltran, W.A.; Aguirre, G.D.; Jacobson, S.G.; Cideciyan, A. V; Lewin, A.S.; Boye, S.L.; Hauswirth, W.W.; Deng, W.-T. AAV-mediated gene therapy for RPGR X-linked retinal degeneration. Patent US9770491B2 2012, 1–40.

67. Deng, W.T.; Dyka, F.M.; Dinculescu, A.; Li, J.; Zhu, P.; Chiodo, V.A.; Boye, S.L.; Conlon, T.J.; Erger, K.; Cossette, T.; et al. Stability and Safety of an AAV Vector for Treating RPGR-ORF15 X-Linked Retinitis Pigmentosa. *Hum. Gene Ther.* **2015**, *26*, 593–602.

68. Smith, A.; Georgiadis, A.; Hoke, J.; Ribeiro, J.; Basche, M.; Robbie, S.; Gonzalez-Cordero, A.;

Ovando-Roche, P.; Naylor, S.; Bainbridge, J.; et al. Efficacy and safety of AAV2/5-hRKp.RPGR to treat X-linked retinitis pigmentosa. ESGCT XXV Anniversary Congress 2017, 1.

69. Fischer, M.D.; McClements, M.E.; Martinez-Fernandez de la Camara, C.; Bellingrath, J.S.; Dauletbekov, D.; Ramsden, S.C.; Hickey, D.G.; Barnard, A.R.; MacLaren, R.E. Codon-Optimized RPGR Improves Stability and Efficacy of AAV8 Gene Therapy in Two Mouse Models of X-Linked Retinitis Pigmentosa. *Mol. Ther.* **2017**, *25*, 1854–1865.

70. Bellingrath, J.-S.E. PhD thesis: Optimising Gene Therapy for X-linked Retinitis Pigmentosa, Universität Tübingen, 2019.

71. Zallocchi, M.; Binley, K.; Lad, Y.; Ellis, S.; Widdowson, P.; Iqball, S.; Scripps, V.; Kelleher, M.; Loader, J.; Miskin, J.; et al. EIAV-based retinal gene therapy in the shaker1 mouse model for usher syndrome type 1B: Development of UshStat. *PLoS One* **2014**, *9*.

72. Truran, R.; Buckley, R.; Radcliffe, P.; Miskin, J.; Mitrophanous, K. Virus purification. Patent US9169491B2 2008, 1–18.

73. Payne, S.L.; Rausch, J.; Rushlow, K.; Montelaro, R.C.; Issel, C.; Flaherty, M.; Perry, S.; Sellon, D.; Fuller, F. Characterization of infectious molecular clones of equine infectious anaemia virus. *J. Gen. Virol.* **1994**, *75*, 425–429.

74. Kong, J.; Kim, S.-R.R.; Binley, K.; Pata, I.; Doi, K.; Mannik, J.; Zernant-Rajang, J.; Kan, O.; Iqball, S.; Naylor, S.; et al. Correction of the disease phenotype in the mouse model of Stargardt disease by lentiviral gene therapy. *Gene Ther.* **2008**, *15*, 1311–1320.

75. Ye, G.J.; Budzynski, E.; Sonnentag, P.; Miller, P.E.; Sharma, A.K.; Ver Hoeve, J.N.; Howard, K.; Knop, D.R.; Chulay, J.D. Safety and Biodistribution Evaluation in Cynomolgus Macaques of rAAV2tYF-CB-hRS1, a Recombinant Adeno-Associated Virus Vector Expressing Retinoschisin. *Hum. Gene Ther. Clin. Dev.* **2015**, *26*, 165–176.

76. Thomas, D.L.; Wang, L.; Niamke, J.; Liu, J.; Kang, W.; Scotti, M.M.; Ye, G.; Veres, G.; Knop, D.R. Scalable rAAV Production Using rHSV Co-infection of Suspension-Adapted Mammalian Cells. *Hum. Gene Ther.* **2009**, *20*, 861–870.

77. Boye, S.L.; Hauswirth, W.W.; Byrne, B.J. Self-complementary adeno-associated virus having a truncated CMV-chicken β-actin promoter. Patent US8298818B2 2006, 1–28.

78. Cukras, C.; Wiley, H.E.; Jeffrey, B.G.; Sen, H.N.; Turriff, A.; Zeng, Y.; Vijayasarathy, C.; Marangoni, D.; Ziccardi, L.; Kjellstrom, S.; et al. Retinal AAV8-RS1 Gene Therapy for X-Linked Retinoschisis: Initial Findings from a Phase I/IIa Trial by Intravitreal Delivery. *Mol. Ther.* **2018**, *26*, 2282–2294.

79. Wright, J.F. Transient transfection methods for clinical adeno-associated viral vector production. *Hum. Gene Ther.* **2009**, *20*, 698–706.

80. Marangoni, D.; Bush, R.A.; Zeng, Y.; Wei, L.L.; Ziccardi, L.; Vijayasarathy, C.; Bartoe, J.T.; Palyada, K.; Santos, M.; Hiriyanna, S.; et al. Ocular and systemic safety of a recombinant AAV8 vector for X-linked retinoschisis gene therapy: GLP studies in rabbits and Rs1-KO mice. *Mol. Ther. Methods Clin. Dev.* **2016**, *5*, 16011.
